# Whey for Sarcopenia; Can Whey Peptides, Hydrolysates or Proteins Play a Beneficial Role?

**DOI:** 10.3390/foods9060750

**Published:** 2020-06-05

**Authors:** Sarah Gilmartin, Nora O’Brien, Linda Giblin

**Affiliations:** 1 Teagasc Food Research Centre, Moorepark, Fermoy, P61 C996 Co. Cork, Ireland; Sarah.Gilmartin@teagasc.ie; 2 School of Food and Nutritional Science, University College Cork, T12 YN60 Co. Cork, Ireland; nob@ucc.ie

**Keywords:** sarcopenia, whey protein, muscle, C2C12, aged animals, older adult, exercise

## Abstract

As the human body ages, skeletal muscle loses its mass and strength. It is estimated that in 10% of individuals over the age of 60, this muscle frailty has progressed to sarcopenia. Biomarkers of sarcopenia include increases in inflammatory markers and oxidative stress markers and decreases in muscle anabolic markers. Whey is a high-quality, easily digested dairy protein which is widely used in the sports industry. This review explores the evidence that whey protein, hydrolysates or peptides may have beneficial effects on sarcopenic biomarkers in myoblast cell lines, in aged rodents and in human dietary intervention trials with the older consumer. A daily dietary supplementation of 35 g of whey is likely to improve sarcopenic biomarkers in frail or sarcopenia individuals. Whey supplementation, consumed by an older, healthy adult certainly improves muscle mTOR signaling, but exercise appears to have the greatest benefit to older muscle. In vitro cellular assays are central for bioactive and bioavailable peptide identification and to determine their mechanism of action on ageing muscle.

## 1. Introduction

From 1980 to 2019, the number of people over the age of 65 worldwide doubled to 810 million people. This populace will reach 2 billion people by 2050 [1]. With a rapidly aging population, there is a need to understand how dietary intervention can counteract the physical impediments of the ageing process on muscle. The reduction in the sum of muscle fibers and size, in parallel with the deficit in spinal motor neurons, results in weakened mechanical muscle ability [2]. This, in turn, affects balance, gait and overall ability to perform tasks of daily living such as rising from a chair unassisted or the ability to walk independently [3]. Our percentage of muscle mass declines after the age of 30 at a rate of 3%–5% every 10 years and this decline accelerates after the age of 60 [4]. A decline in muscle mass and strength may eventually result in an individual presenting with a muscle mass lower than two standard deviations of the adult population mean and having a gait speed of <0.8 m/s [5]. At this juncture, a clinical diagnosis of sarcopenia is made [6]. By 2045, the incidence of sarcopenia in Europe will increase from 19 m (2016 figures) to 32 m—a 68% increase [7]. Shafiee et al. [8] estimated the incidence of sarcopenia in adults over the age of 60 at 10% based on the assessment of 58,404 individuals using the European Working Group on Sarcopenia in Older People (EWGSOP) [9], the International Working Group on Sarcopenia (IWGS) [10] and the Asian Working Group for Sarcopenia (AWGS) [11] definitions. A dual-energy X-ray absorptiometry scan (DEXA) is the preferred method used in the diagnosis of sarcopenia [12]. It evaluates fat mass together with bone mass, albeit its inability to decipher between water retention and fat infiltration in muscle can result in an 8% overestimation of skeletal muscle [13]. Other methods used to measure muscle mass include, bioelectrical impedance, neutron activation assessments and urinary excretion of creatinine [6]. In muscle cells, creatine to phosphocreatine acts as an important phospho energy store and is mediated by creatine kinase [14]. Since 90% of the body’s phosphocreatine is stored in muscle tissue [15], circulating levels of its breakdown product, creatinine, is regarded as a reliable biomarker for muscle mass (where kidney function is normal) [16]. For 82 sarcopenic individuals, Rong et al. [17] observed that serum creatinine levels (66.68 ±14.21 μmol/L vs. 73.16 ±11.73 μmol/L, *p* < 0.05) were significantly lower than 82 non-sarcopenic individuals of similar age. Although further analysis with the dataset using univariate regression only indicated a tendency to associate with sarcopenia (*p* = 0.058), other studies have also observed significant associations between the serum creatinine pathway and sarcopenia [18,19].

### 1.1. Sarcopenia Associated Blood Biomarkers-Inflammatory Cytokines, Hormones, Muscle Anabolic Signals and Oxidative Stress Indicators

Although blood biomarkers are not used to diagnose sarcopenia, recent studies have shown significant differences in a range of other blood biomarkers between control and sarcopenic individuals of similar ages. Not surprisingly, circulating levels of several inflammatory cytokines have been associated with sarcopenia, indicating an inflammasome role in sarcopenia [20]. Most notably, interleukin-6 (IL-6) was significantly higher in those diagnosed with sarcopenia [17,18,21] with Rong et al. [17] reporting serum levels of 43.80 ± 10.13 pg/mL (*n* = 82) compared to 27.38 ± 9.53 pg/mL in the control group (*p* < 0.05) and Bian et al. [18] reporting 49.77 ± 22.14 pg/mL serum IL-6 in 79 individuals vs. 39.72 ± 29.53 pg/mL in the control group (*p* = 0.03). Univariate logistic regression analysis highlighted increased IL-6 as a risk factor for sarcopenia [21], with an increase in plasma IL-6 associating with a slower walking speed in the older adult (*n* = 854 mean age of 74.3 ± 2.7 years) [22]. However, it is controversial, with a meta-analysis performed on 17 studies with 3072 sarcopenic individuals reporting that serum IL-6 levels were similar in sarcopenic and non-sarcopenic participants [23]. Interleukin 10 (IL-10) is also regarded as a biomarker of note with elevated levels in serum of sarcopenic individuals (4.13 ± 1.03 pg/mL compared to 3.75 ± 1.21 pg/mL) compared to the control group [17]. However, Kwak et al. [21] observed no differences in plasma IL-10 levels in 50 sarcopenic vs. 46 non-sarcopenic individuals and Stowe et al. [24] reported no increase with age in 1411 individuals. In the study by Bian et al. [18], Tumor Necrosis Factor-α (TNF-α) was significantly elevated in serum collected from sarcopenic individuals (165.39 ± 19.49 pg/mL) compared to controls (148.79 ± 26.06 pg/mL, (*p* = 0.01)). In vastus lateralis biopsies, TNF-α mRNA transcript was 2.8 fold higher in older men (*n* = 16, 70 years old) compared to 13 men aged 20 [25], albeit the older individuals were not diagnosed with sarcopenia. Plasma concentrations of TNF-α do indeed increase as we age [24] but Kwak et al. [21] did not observe differences in sarcopenic vs. non-sarcopenic individuals over the age of 60 and a meta-analysis study by Bano et al. [23] study did not identify TNF-α as a biomarker of interest. The acute phase protein C-reactive protein (CRP), however, has received some considerable attention. Bano et al. [23] stated that 3072 sarcopenic individuals had significantly higher levels of circulating CRP (SMD = 0.51; 95%CI 0.26, 0.77; (*p* < 0.0001); I2 = 96%) than 8177 controls. Taaffe et al. [22] associated increased levels of this hepatic inflammatory protein in serum with reduced grip strength. The pro-inflammatory cytokine macrophage migration inhibitory factor (MIF) was also identified by Kwak et al. [21] as one of four biomarkers from a total of 21 blood biomarkers that could be used in a screening panel for sarcopenia with significantly higher levels in the sarcopenic participants (25.1 ± 1.19 vs. 20.71 ± 0.89 ng/mL, (*p* = 0.008)). The four biomarkers were IL-6, MIF, an extracellular matrix repair glycoprotein secreted protein acidic and rich in cysteine (SPARC), and insulin-like growth factor 1 (IGF-1). IGF-1 involvement in sarcopenia may not be a surprise as IGF-1 plays an important role in muscle protein synthesis and is known to decrease with age [26]. IGF-1 participates in muscle anabolism via Akt phosphorylation activating the mammalian target of rapamycin (mTOR) pathway which ultimately controls muscle protein synthesis and turnover [27]. Sarcopenia results from a decrease in muscle anabolic pathways with an increase in catabolic pathways [28]. For sarcopenic individuals, Kwak et al. [21] observed a significant (further) decrease in serum IGF-1 from 72.61 ± 5.49 ng/mL to 58.16 ± 3.37 ng/mL compared to the non-sarcopenic control group of similar age. Oxidative stress due to free radical damage has been suggested as one of the most prominent causes of skeletal muscle reduction that occurs with ageing [29]. These radicals are exceedingly reactive species with the ability, either in the nucleus or in cell membranes, of damaging DNA, proteins, carbohydrates and lipids [30]. To mop up free radicals and maintain redox homeostasis, the muscle cell employs enzymes such as glutathione peroxidase (GPx) [31], superoxide dismutase (SOD) [32] and catalase (CAT) [33]. Studies have shown that oxidative damage biomarkers, 8-hydroxy-2′deoxyguanosine (8-OHdG) and malondialdehyde (MDA) are increased in skeletal muscle as we age [34].

### 1.2. Why Whey?

Skeletal muscle (SM) makes up approximately 40% of total body weight, consisting of 50%–75% of total proteins, and is accountable for 30%–50% of total body protein turnover [35]. Dietary intervention with a high-quality protein could modulate the biomarkers of ageing muscle and/or delay the onset of sarcopenia [36]. Bovine whey is used, in a wide variety of products (beverages and protein bars), in the sports nutrition market to promote muscle growth [37] and repair [38] after physical exercise [39]. Its success is predicted to be due to the fact that whey proteins (1) are easily digestible [40,41], (2) contain all essential amino acids [42], (3) are a rich source of branch-chain amino acids (BCAA) [43] which activates the mTOR pathway [44,45], and (4) are a rich source of bioactive peptides [46]. Bovine whey is composed of β-lactoglobulin (50%–60%), α-lactalbumin (15–25%), bovine serum albumin (BSA, 6%), lactoferrin (<3%) and immunoglobulins (<10%) [47]. Bovine whey is used in food formulation in different formats that differ in their protein concentration and degree of protein hydrolysis; i.e., liquid whey, whey protein isolate, whey protein concentrate or whey protein hydrolysate [48].

This review considers the evidence that whey peptides, hydrolysates, proteins or products can delay or reduce symptoms of sarcopenia or alter biomarkers of sarcopenia in the older adult, in aged animals or muscle cells lines.

## 2. Whey Peptides on Muscle Cell Lines In Vitro

Treatment of murine myoblast cell line, C2C12, with whey peptides, hydrolysates or intact protein is summarised in Table 1. Previously, our group [49] has identified several whey peptides that are produced during simulated upper gastrointestinal digestion of whey protein isolate. A subgroup of these peptides was capable of crossing the intestinal barrier, as evidenced by their appearance on the basolateral side of Caco2-HT29 monolayers. Within this subset, four peptides ALPM, GDLE, VGIN and AVEGPK (5 mM) reduced oxidative stress in undifferentiated C2C12 cells [49]. Whether these peptides can be detected in plasma after dietary intervention with whey is as yet unknown. Ogiwara et al. [50] identified a dipeptide (MH), wheylin-1, from β-lactoglobulin produced during thermolysin enzymatic hydrolysis. This peptide significantly increased insulin-induced Akt phosphorylation in differentiated C2C12 cells compared to insulin induction alone or compared to control cells. Whether this peptide can cross the intestinal barrier is unknown. Certainly, intraperitoneal injection of wheylin-1 (1 mg/kg body weight) in young mice (*n* = 5) increased Akt phosphorylation in skeletal gastrocnemius muscle. Its effect in dietary intervention trials on aged animals or older/sarcopenic humans has not been tested. Mobley et al. [51] differentiated C2C12 cells prior to treatment with whey hydrolysate and surprisingly noted a significant decrease in mRNA transcripts of the mTOR biomarker, raptor (a regulatory associated protein [52]), compared to levels in cells in DMEM media alone. The peptide or amino acid composition of the whey hydrolysate was not given. Kerasioti et al. [53] pre-incubated differentiated C2C12 cells with sheep whey protein for 24 h and then oxidatively stressed the cells with tertbutyl hyrdoperoxide for 30 min. Glutathione (GSH) levels significantly increased and ROS decreased with treatment of sheep whey protein at 1.56, 3.12 and 6.24 mg/mL, albeit the description (intact/hydrolysate) of the whey protein was not provided. Xu et al. [54] also measured levels of GSH, SOD, CAT and G-Px to evaluate the protective effects of whey protein on undifferentiated C2C12 cells from oxidative damage. Cells pre-treated with whey protein for 24 h and then subjected to hydrogen peroxide stress, resulted in significant decreases of MDA levels and significant increases in GSH, SOD, CAT and G-Px levels compared to hydrogen peroxide stressed cells. Knight et al. [55] purified the minor protein, Ribonuclease 5, from bovine whey and demonstrated that this purified fraction (95% pure) not only significantly increased myogensis of C2C12 cells over a 4-day period but also increased C2C12 creatine kinase activity. Most notably, these increases were similar to IGF-1 treatment. However, it is important to question the rational for applying intact whey proteins directly to muscle cells as only a limited number of whey peptides will survive the proteolytic conditions of the gut and cross the intestinal barrier to reach muscle. Knight et al. [55] did report a dietary intervention trial in adult, but not aged mice, where grip strength and muscle weight were significantly increased after 3 weeks and 4 months, respectively, with a diet supplemented with Ribonucelase 5 (50% purity, 17 ug/g feed). Indeed, Carson et al. [56] collected serum from six healthy men 60 min after ingesting whey protein hydrolysate (0.33 g/kg body weight). C2C12 myotubes were then treated with this serum in the presence of media for 4 h. Phosphorylation to total protein ratios were increased for mTOR, p70S6K, 4EBP1 in media conditioned with serum post whey consumption vs. serum from fasted state. This increased phosphorylation status was not observed when cells were incubated with serum collected from individuals who consumed an equivalent non-essential amino-acid-based beverage.

## 3. Effects of Whey Products on Aged Animal Models

Rats and mice over the aged of 24 months are considered equivalent to humans of >60 years of age [57,58]. Recently, several intervention trials with whey have been performed on aged rodents, (Table 2). Many of these studies included whey dietary intervention in combination with BCAA, leucine or with potent antioxidants. Van Dijk et al. [59] fed 22-month-old mice for 3 months with a whey-based diet or a whey-based diet supplemented with antioxidants selenium, zinc, vitamin A and vitamin E. Dietary intervention with whey or antioxidants had no effect on lean mass compared to control group but in combination they significantly increased lean body mass. Maximal in vivo muscle strength was significantly increased in mice fed whey or whey plus antioxidants compared to control diets. Nocturnal physical activity was higher in the whey treatment group. Mosoni et al. [60] compared two different doses of whey-based diets, whey protein (12%) or (18%) with/without anti-inflammatory/antioxidant mix using 16-month-old rats over a period of 6 months. Lean body mass % loss associated with ageing was less with 18% whey supplementation than with 12% whey. There was no difference in vivo muscle fractional synthesis rate across whey dosage or casein control diets. Ex vivo protein synthesis rate and proteolysis rate were measured in biopsied epitrochlearis muscle. Ex vivo synthesis rate with whey was significantly increased over casein diet and there was no difference with ex vivo proteolysis rate. Supplementation with anti-inflammatory/antioxidant mix in both diets significantly reduced the redox biomarker, thiobarbituric acid reactive substances in muscle, while increasing muscle GSH and plasma antioxidant activity. In contrast, casein diet-improved liver SOD levels compared to whey. Plasma fibrinogen, an inflammatory biomarker was elevated after 6 months in all groups compared to time zero. The authors viewed this increase as an indicator of ageing in the animals. The higher dose of whey provided a protective effect from this ageing-associated inflammation, with fibrinogen significantly less in the group fed 18% whey compared to those fed 12% but not casein. Garg et al. [61] investigated the ability of whey protein concentrate to counteract the effects of oxidative stress in 24-month-old aged male rats. Rats were fed whey protein concentrate orally for 28 days. Erythrocytes were harvested and structural and functional integrity of their cell membranes examined. Membrane integrity deteriorates with age with notable decreases in sialic groups and increases in carbonyls and lipid peroxidation. Garg et al. [61] observed that the membrane sulfhydryl groups and sialic acid groups were significantly increased, whereas lipid hydroperoxide and protein carbonyls were both significantly decreased in erythrocytes membranes of old rats fed whey protein concentrate in comparison to aged controls.

Dijk et al. [62] favored the combination of whey protein isolate with leucine in a post-prandial study with 25-month-old mice. The rate of muscle protein synthesis was measured in response to oral gavage with whey protein isolate plus leucine compared to oral gavage with leucine or a fasted control. Interestingly, the whey and leucine combination resulted in a significant increase in muscle protein synthesis after 60 min post oral gavage in comparison to the control groups. Phosphorylation of Akt and other mTOR signalling proteins (4E-BP1 and p70S6k) were significantly elevated at 60 min post-ingestion of whey and leucine compared to leucine alone (Akt, 4E-BP1) or the fasted state (Akt, 4E-BP1 and p70S6k). Jarzaguet et al. [63] fed aged rats (20 months) whey proteins 144 g/kg feed containing leucine 16.2 g/kg. Phosphorylation status of Akt and other mTOR biomarkers (S6K1 and S6) were significantly elevated after ingestion of whey bolus compared to casein or soy with some diet by time interactions. Muscle protein synthesis rate was also significantly increased compared to casein intervention with time by diet interactions noted with soy diet. Walrand et al. [64] examined the effects of whey protein on muscle weight, strength and protein synthesis in ad libitum diets, protein-restricted or energy-restricted diets in 21-month-old aged rats for 5 months. Whey protein enhanced muscle absolute synthesis rate for all three diets. However, only when diets were restricted did whey intervention have a tendency to have a positive effect on skeletal muscle strength. There was no significant difference noted between the weights of the soleus muscle across the groups but the muscle weight tended to be higher in the ad libitum whey protein group compared to the ad libitum casein group (266.5 mg vs. 238.7 mg, respectively) [64]. Interestingly, Lafoux et al. [65] favored a combination of whey with exercise in their study on aged rats. Seventeen-month-old rats were fed a bolus of 0.85 g of whey or casein or milk protein and were subjected to either a sedentary or active routine over a 2-month period. Although there were some notable differences compared to time zero (Table 2), total movement, distance travelled activity time or average speed did not differ between whey or treatment groups. Interestingly, Magne et al. [66] focused on muscle recovery in the older animal. The experimental design included immobilisation of the hind limbs of aged rats (22–24 months) for 8 days. Upon casting removal, the animals were fed for 40 days on a diet which contained whey protein at 144 g/kg feed. Muscle mass gain in animals that received whey was significantly increased by ~200 mg by day 20 and further increased to ~400 mg by day 40 compared to casein- or leucine-rich diet groups. An increase in postprandial muscle protein synthesis and amino acid concentration were both noted after 40 days with whey compared to a casein diet, to day 1 of recovery or to a leucine-rich diet.

All of the rodent studies observed a beneficial effect of whey supplementation on one or more biomarkers of interest (e.g., antioxidant, muscle protein synthesis and muscle Akt phosphorylation levels). In terms of dosage, it is interesting to note that in murine studies, mice received 136 g whey per kg feed, which equates to 0.54 g whey per day, assuming an older mouse consumes approximately 4 g feed. In studies with aged rats, whey dosage was 140–215 g whey per kg feed, which equates to 2.9–4.5 g whey/day if an older rat consumes 21 g feed per day [60]. Bolus and oral gavage experiments were performed with 0.85, 0.864 or 0.15 g whey for rats and 0.139 g whey for mice. In addition, animal trials were performed with whey protein isolate, whey protein concentrate or undefined whey with no mention of whey hydrolysates or individual whey peptides.

## 4. Intervention Trials with Whey and the Older Adult

In humans, a number of postprandial studies (Table 3) with whey supplementation have been performed in the older adult. The direct method to track the fate of whey post-ingestion is to label it. Pennings et al. [67] investigated the effects of radiolabelled whey protein in 33 healthy, elderly men aged 73 ± 2 yrs. An infusion of L-[1-^13^C] phenylalanine to a lactating Holstein cow resulted in radiolabelled milk from which the whey protein fraction was purified. The 73-year-old man received 10, 20 or 35 g of this radiolabelled whey. At 240 min, muscle biopsies were collected from the vastus lateralis and muscle tissue analysis was performed. Whole body protein breakdown was significantly decreased in the groups post whey protein consumption when compared to time zero. Synthesis, oxidation and net balance of proteins were all significantly increased compared to time zero after ingestion of 35 g of whey protein. Overall, net balance of protein metabolism was significantly higher for the 35 g whey protein supplemented group compared to the 20 g whey protein group. Muscle biopsies also revealed dose-dependent increases in muscle fractional synthesis rate and incorporation of radiolabelled phenylalanine. Reitelseder et al. [68] performed a postprandial study combining a 300 mL whey protein hydrolysate beverage (0.45 g/kg) with exercise over a 6 h period in older men (aged 61 ± 1 year) (*n* = 10). A continuous infusion by arm vein catheterization of radioactive free L [^15^n]-phenylalanine tracer allowed for protein-bound phenylalanine measurement in biopsied vastus lateralis muscle. Whey increased muscle protein synthesis compared to basal control but was similar to casein or carbohydrate intervention. In addition, mTOR phosphorylation status was unaffected in this acute study. With limited effects reported, the authors questioned whether or not older muscle would respond to an increase in dietary intake of whey protein. Borack et al. [69] combined whey protein isolate with exercise and examined the effects of 30.4 g of whey protein isolate in elderly individuals (55–75 years of age) on phosphorylation of mTOR biomarkers and fractional protein rates in vastus lateralis biopsies. Although consumption of whey increased muscle mTOR phosphorylation, increased fractional protein synthesis and decreased breakdown compared to baseline, there was no difference with consumption of a soy-dairy protein blend. Wilkinson et al. [70] compared the effects of whey protein (40 g bolus) with or without exercise on muscle protein synthesis in older women aged 65 ± 1 year (*n* = 24) in a 7 h postprandial study. Muscle myofibrillar protein fractional synthesis rate (FSR) significantly increased after 2 h with whey supplementation plus exercise compared to time zero but was similar to the 6 g of leucine plus exercise group. Interestingly, phosphorylation of mTOR protein, p-p70S6K1 was significantly increased with whey compared to time zero. However, exercise was required to increase phosphorylation of p70S6K1 in both the leucine and whey treatment groups vs. time zero. Kramer et al. [71] performed a postprandial study to investigate the effects of leucine-enriched whey protein supplementation (3 g of leucine/21 g of whey) in healthy (69 ± 1 years old) (*n* = 15) and sarcopenic (81 ± 1 years old) (*n* = 15) males. This study also included a continuous infusion by arm vein catheterization of radioactive free L-[ring-^15^C_6_]-phenylalanine tracer to allow for protein-bound phenylalanine measurement in muscle (vastus lateralis). Muscle protein-bound enrichment gain was higher in the sarcopenic group at time zero after ingestion of whey protein and increased significantly in both sarcopenic and healthy control groups over time. Mixed muscle protein synthesis rate was significantly increased postprandially compared to basal values following whey protein supplementation, regardless of whether an individual was sarcopenic or healthy. Dideriksen et al. [72] examined the effects of 0.45 g/kg LBM whey protein isolate plus resistance training in 14 older men aged ≥ 60 years of age with a high level of plasma CRP (>2 mg/L). Seven subjects were given 1800 mg/day of ibuprofen in tandem with whey and [^15^N] phenylalanine stable isotope tracer. A further seven subjects received whey protein and a placebo tablet containing potato starch and lactose monohydrate. Myofibrillar FSR was elevated following intake of whey protein in both resting and exercise legs in comparison to time zero. Myofibrillar FSR was higher in the exercised leg compared to the resting leg. There was a significant difference for all three groups for basal, postprandial and post-exercise states but there was no difference between the control (untreated), whey or whey plus ibuprofen groups themselves. For those in the whey plus exercise groups, muscle connective tissue was increased compared to time zero. Smith et al. [73] measured phosphorylation status of mTOR^Ser2448^, AKT^Ser473^, and AKT^Thr308^ proteins in 22 women (aged 57.8 ± 4.2 years) during a hyperinsulinemic-euglycemic clamp procedure with/without whey protein. Phosphorylation of mTOR^Ser2448^ and p70S6K^Thr389^ were greater during whey protein supplementation compared to Kool Aid controls and AKT^Ser473^ and AKT^Thr308^ were increased with whey compared to time zero.

Exercise, specifically resistance or strength training has been shown to increase muscle mass in the elderly [74]. Whether whey in combination with resistance training (Table 4) can increase muscle mass and strength in a bid to combat sarcopenia has been investigated by a number of research groups. Nabuco et al. [75,76] examined the effects of 35 g of hydrolysed whey protein supplementation in older women aged > 60 years old over a 26-week period (*n* = 47). Whey protein consumption resulted in a decrease in uric acid compared to the maltodextrin group. Whey increased SOD, CAT and total radical-trapping antioxidant parameter (TRAP) vs. time zero. Whey resulted in a decrease in advanced oxidation protein products (AOPP) and lipid hydroperoxide compared to time zero. Whey supplementation resulted in a decrease in the 10 min walk speed test compared to time zero and maltodextrin placebo. A decrease in the time taken to rise from a seated position following whey consumption was also recorded compared to time zero. Whey supplementation resulted in an increase in knee extension, chest press and total strength compared to time zero and maltodextrin placebo. An increase in the arm exercise preachers curl was also noted following whey vs. time zero. Percentage of skeletal muscle mass was significantly increased following whey protein consumption compared to maltodextrin placebo. To try to unravel exercise from whey intervention, Sugihara Junior et al. [77] included a pre-conditioned 8-week resistance training followed by a 12-week whey intervention plus resistance training with women aged 67.4 ± 4 years old. The group supplemented with hydrolysed whey protein exhibited a significantly greater increase in chest presses, knee extensions, and total strength compared to maltodextrin placebo plus exercise and compared to 8-week exercise alone. Mori et al. [78] investigated the effects of 22.3 g of whey protein with and without exercise over a 24-week period in healthy elderly woman aged 65–80 years old (*n* = 75). Exercise (rising and sitting from a chair, plus leg extension with resistance band exercises) with whey was better than exercise alone which, in turn, was better than whey alone for muscle mass, grip strength, knee extension and gait speed. Interestingly, any intervention significantly improved these markers from time zero measurements. Englund et al. [79,80] examined the effects of whey protein supplementation (20 g) with Vitamin D (800 IU) and resistance training over a 6-month period in older adults aged 78.5 ± 5.4 years old (*n* = 149). Whey consumption with exercise resulted in an increase in normal density muscle compared to control (a low-calorie placebo drink 30 kcal, no protein, no Vitamin D) and exercise group. There was a significant decrease in low-density muscle in the whey protein group compared to baseline parameters and normal muscle density. Both whey plus exercise and control plus exercise groups showed a significant increase in knee flexor strength, power and quality compared to time zero. Whey protein supplementation and exercise resulted in a greater decline in intramuscular fat compared to the control plus exercise group. Chalé et al. [81] analyzed the effects of whey protein concentrate supplementation (40 g/daily) in 42 elderly adults (aged 70–85 years old) with resistance training for 6 months. Knee extensor power was the only significant increase following whey consumption compared to the maltodextrin control group. There was an observable increase in total mid-thigh cross-sectional area (CSA), total muscle CSA and total normal density muscle CSA in the whey protein group compared to time zero but no difference compared to maltodextrin. Kirk et al. [82] investigated the effects of leucine (0.03 g/kg/meal) enriched whey protein (0.5 g/kg/day) in elderly individuals (aged ≥60–86 years old) over a 16-week period in conjunction with exercise (*n* = 46). Significant increase in leg press, chest press and bicep curl were observed from pre- to post-intervention in both the whey protein in combination with exercise and exercise only groups. Ingestion of whey did not increase all fore-mentioned parameters over and above exercise alone, albeit whey plus exercise was better than time zero. In a follow-on study [83], rectus femoris and bicep femoris were less fatigued with exercise intervention but no additional improvement with whey compared to time zero. No differences were noted for muscle mass or handgrip between or within whey and exercise, exercise only, whey only or control (untreated) groups. Exercise alone appears to have a more profound effect on muscle health compared to whey and exercise or even whey alone. Hospitalized individuals are particularly vulnerable to muscle wastage. Whether whey protein supplementation can preserve muscle mass in this cohort was investigated by Niccoli et al. [84]. Frail patients aged 81.3 ± 1 years old (*n* = 47) were supplemented with 24 g of whey protein per day by incorporation in their porridge (9 g) and the remaining via dairy beverages (7.5 g whey/drink) at lunch and dinner. There was a significant improvement in grip strength (30.3%) and knee extensor force (42.7%) compared to time zero. Gait speed, and time to ‘get up and go’ for those supplemented with whey protein was significantly improved compared to pre-intervention parameters. Serum IL-6 was shown to significantly decrease following whey protein supplementation compared to time zero for both the whey-protein-supplemented group and the control group (hot cereal and milk products). Whey resulted in a greater decrease in percentage IL-6 compared to the control group (hot cereal and milk products without whey protein). Whether whey protein supplementation can aid in enhancing physical function following discharge from hospital was investigated by Gade et al. [85,86]. They examined the influence of whey protein supplementation and resistance training in older adults aged 70 years or older (*n* = 141) during their hospital stay and 12 weeks after discharge. Subjects were given a ready-to-drink milk-based protein supplement containing 27.5 g whey protein/day in combination with a resistance training programme over a 12-week period. Overall, there was no noted benefit (30 s chair stand test, hand grip strength, 4 m gait speed) of whey protein supplementation in this study. Mancuso et al. [87] investigated the effects of a whey-based oral supplement (10 g/day for 30 days) in 13 individuals suffering from mitochondrial disease aged 52.5 ± 15.2 years. Defective mitochondria can trigger a series of events that results in death of motor neuron and muscle fiber death, ultimately causing sarcopenia. Following treatment with whey advanced oxidation protein products (AOPP), a biomarker of oxidative stress in plasma was significantly decreased, while plasma FRAP and GSH were significantly increased during resting compared to the casein placebo group and baseline parameters.

Table 5 describes intervention trials in the older adult with whey supplementation where exercise was not included. Chanet et al. [88] reported on the effects of leucine-enriched whey protein (21 g) in combination with vitamin D (800 IU) in 12 healthy older men (71 ± 4 years old) over a 6-week period. Infusion by arm vein catheterization of radioactive free L-[^2^H_5_]-phenylalanine tracer was performed to allow for protein-bound phenylalanine measurement in muscle (vastus lateralis). Mixed muscle protein synthesis rate was elevated compared to the controls (flavored water) and to time zero. There was a significant increase in appendicular muscle mass and leg lean mass in the whey protein group compared to the flavored water group after 6 weeks. There was no difference noted between the whey protein group or the flavored water group for inflammatory biomarkers. Rodondi et al. [89,90] investigated the effects of whey protein supplementation (15 g) in combination with essential amino acids (5 g) with or without zinc (30 mg/day) in 47 elderly patients aged 85 ± 7.4 years for 4 weeks. With whey protein supplementation, activity of daily living score improved marginally suggesting a level of independent living by the subject. In the whey protein groups, IGF-1 levels were significantly increased vs. the controls (untreated). Coker et al. [91] examined the effects of a calorie-restricted whey-based meal replacer (170 kcal/ 5 time/day). Eleven obese elderly subjects aged 65–80 years consumed a whey protein (7 g) and essential amino acid (EAA) (6 g) meal replacement. There was an increase in skeletal muscle FSR in the whey group which was significantly different from the control group (competitive meal replacer).

Studies describing whey supplementation with whey protein on sarcopenic individuals are detailed in Table 6. In longer term studies, Nabuco et al. [92] investigated the effects of whey in 26 elderly women > 60 years of age, with sarcopenic obesity, who consumed 35 g hydrolysed whey protein over a 16-week period in tandem with resistance training (chest press, horizontal leg press, seated row, knee extension, preacher curl, leg curl, triceps pushdown, seated calf raise). Whey protein significantly reduced circulating IL-6 compared to maltodextrin and time zero. However, the vast majority of sarcopenic biomarkers were improved by exercise rather than whey. For instance, at the end of the trial, there was a significant reduction in the number of individuals defined as sarcopenic, and a significant decrease in circulating TNF-α and CRP with an increase in blood antioxidant potential compared to time zero but this was similar for whey or maltodextrin. Liberman et al. [93] investigated the effects of a 13-week supplementation programme of vitamin D (800 IU) and leucine-enriched whey protein (3 g leucine/20 g WP) on the chronic low-grade inflammatory profile (CLIP) in sarcopenic people (aged ≥ 65 years *n* = 297). For plasma IL-6, a treatment x time interaction was noted, with no significant difference over time for the whey group compared to a significant increase for the control group (31.4 g of carbohydrates). There was no significant difference in plasma IL-1Ra or IL-8 following whey protein supplementation. Bo et al. [94] investigated the effects of whey protein supplementation (57.5% protein) in combination with vitamin D (702 IU) and E (109 mg) on sarcopenic individuals aged 60–85 years old (*n* = 60). After 6 months of whey protein supplementation, appendicular muscle mass, relative skeletal muscle mass index, handgrip strength and serum IGF-1 were all increased compared to the isocaloric placebo (32.4 g of carbohydrates, 2.6 g of fat). In addition, there was a significant decrease in serum IL-2 and in the time taken to perform a ‘time to stand test’ in comparison to the placebo group. Li et al. [95] compared the supplementation of whey protein in tandem with resistance training in sarcopenic individuals (72.05 ± 6.54 years old) to non-sarcopenic individuals (65.24 ± 4.05) (*n* = 56 per group) over a 12-week period. The whey protein supplement contained 30 g of whey protein and 3.84 g of leucine per serving taken twice daily. Following whey protein intervention in sarcopenic individuals, there was a significant increase in muscle mass and IGF-1 compared to time zero. There was a significant decrease in TNF-like weak inducer of apoptosis (TWEAK), TNF-α and IL-18 compared to pre-intervention parameters [95]. The PROVIDE study carried out by Hill et al. [96] aimed to investigate the effects of leucine (3 g) enriched whey protein (20 g) supplementation in elderly sarcopenic individuals aged > 65 years (*n* = 380) over a 13-week period. Serum IGF-1 was significantly increased in the whey protein supplementation group compared to the isocaloric control. For the same cohort of individuals, Bauer et al. [97] noted that appendicular muscle mass, hand grip strength, chair stand test time, short physical performance battery score and gait speed were significantly improved in the whey protein group compared to time zero. However, the improvement in several of these biomarkers (hand grip strength, gait speed, SPPB, chair-stand time and balance test) were similar to that observed in the isocaloric control group [96,97].

Although the trials used different scores to classify sarcopenic individuals [95,96], overall whey supplementation improved sarcopenic biomarkers, with a somewhat clearer outcome emerging than data from healthy individuals of similar age. The majority of studies in human trials lasted 4 months [92], with a dosage range of 20–35 g [93,94,95] whey protein per day. There were a number of notable limitations to the human studies. First and foremost was the omission of power calculations in the description of experimental design in 14 trials. In addition, a number of studies including Niccoli et al. [84], Mancuso et al. [87], Reitelseder et al. [68], and Borack et al. [69] had whey in their control group, which confounded the results and made interpretation difficult. Twelve studies included a combination of whey with exercise, making it difficult to unravel the contribution of whey alone without appropriate controls. Many studies observed significant differences to time zero instead of isocaloric [85,96], maltodextrin [75,76,77] or matched protein [68,87] control groups. In addition, many of the studies employed different whey products or ingredients which also made comparisons difficult.

## 5. Conclusions

In summary, a daily dietary supplementation of 35 g whey is likely to improve sarcopenic biomarkers in frail or sarcopenic individuals. For the older healthy adult, whey supplementation certainly improves muscle mTOR signaling, but the greatest benefit to the older muscle is exercise. In vitro cellular assays will continue to play an essential role in identifying bioactive and bioavailable peptides from whey and in unraveling their mechanism of action on the ageing muscle. Future studies should also investigate emerging biomarkers to assess association with sarcopenia, e.g., isoprostanes [98,99] or allantoin [100] as indicators of oxidative stress.

## Figures and Tables

**Table 1:** Effects of whey protein supplementation on muscle cells in vitro. BSA: Bovine serum albumin; ABAP: 2,2′-azobis(2-methylpropionamidine) dihydrochloride; Akt: protein kinase B; DMEM: Dulbecco’s Modified Eagle Medium; tBHP: tert-butyl hydroperoxide; GSH: glutathione; ROS: reactive oxygen species; H_2_O_2_: hydrogen peroxide; MDA: malondialdehyde; SOD: superoxide dismutase; CAT: catalase; G-Px: glutathione peroxidase; IGF-1: insulin-like growth factor 1.

Whey Protein Description	Experimental Detail	Outcome	Reference
ALPM (β-lactoglobulin 142-145AA) GDLE (β-lactoglobulin 52-55AA) VGIN (α-lactoalbumin 99-102AA) AVEGPK (BSA 568-573AA)	Undifferentiated C2C12,-5 mM synthesized peptide pretreated for 1 h, 37 °C prior to ABAP radical (600 μM)	Each peptide ↓ % free radical formation similar to control V cells treated with ABAP treatment	[49]
Wheylin-1 (MH), thermolysin hydrolysis β-lactoglobulin	Differentiated C2C12, 500 μM synthesized peptide for 3 h at 37 °C, plus insulin (100 nM) for 15 min	↑ phosphorylated Akt: Akt total protein V cells treated with insulin alone or V saline	[50]
Hydrolysed whey protein (COMBAT-MusclePharm, proprietary blend from whey protein concentrate)	Differentiated C2C12, 6 h treatment with 13 μg/mL	↓ raptor mRNA V cells treated with DMEM media alone	[51]
Sheep whey protein	Preincubated differentiated C2C12 cells with sheep whey protein for 24 h at 0.78 1.56, 3.12 and 6.24 mg/mL. Stressed with tBHP for 30 min	↑ GSH, ↓ ROS with 1.56, 3.12 and 6.24 mg/mL V cells treated with tBHP alone	[53]
Whey protein	Undifferentiated C2C12 whey protein (0.4 mg/mL) for 24 h and then stressed with 0.5 mM H_2_O_2_	↓ MDA V cells treated with H_2_O_2_ control ↑ GSH, SOD, CAT, G-Px V cells treated with H_2_O_2_ control	[54]
Ribonucelease5 enriched whey from bovine skim milk	Differentiated C2C12,10 μg/mL Ribonuclease 5 fractions that differed in purity, 4-day differentiation	↑ myogenesis myosin heavy chain staining V cells treated with media alone ↑ creatine kinase activity similar to IGF-1 (200 ng/mL) positive control at 3 and 4 days V cells with media alone	[55]

**Table 2:** Aged animal intervention trials outlining the effects of whey supplementation. SOD: superoxidase dismutase; GSH: glutathione;FSR: fractional synthesis rate; Akt: protein kinase B; FFM: fat free mass.

Whey Dosage, Source, Duration	Animal Model	Outcome of Whey Intervention	Reference
Whey (136 g/kg feed) + leucine (16.8 g/kg feed) 3-month intervention Isocaloric diets	22-month-old maleC57/BL6J mice *n* = 8	Whey: No difference lean body mass, ↑ fore limb strength V diet low in antioxidants, ↑ nocturnal physical Activity V control diet ↓ fatigue V control diet, tendency (*p* < 0.07) for improved muscle quality (grip strength/lean mass) V diet low in antioxidants	[59]
Whey protein (140 g/kg feed)(12%) or (215 g/kg feed)(18%) with/without anti-inflammatory /antioxidants mix (chamomile extract 17 g/kg feed, Vitamin E 300 UI/Kg feed and Vitamin D 5000 UI/Kg feed) 6 months	16-month-old male Wistar rats *n* = 11	Whey: No difference in vivo gastrocnemius muscle fractional synthesis rate synthesis or degradation rates Lean body mass loss through ageing ↓ with whey over time 12% and 18% ↑ ex-vivo epitrochlearis muscle synthesis rate V casein ↓ SOD in whey V casein diet ↑ GSH liver in whey + antioxidants V whey - antioxidants ↓ plasma fibrinogen whey 18% V whey 12% but not to casein ↑ plasma antioxidant activity whey V casein diets No difference in vivo muscle FSR across whey dosage or casein control diets Ex vivo synthesis rate with whey was ↑ V casein diet	[60]
Whey protein concentrate (Camillotek), 0.300 g/kg body weight 28 days by oral gavage daily	24-month-old male Wistar rats *n* = 6	Whey:↑ erthyrocyte membrane sulfhydryl groups and sialic acid groups V saline oral gavage ↓ lipid hydroperoxide and protein carbonyls in erthyrocyte membrane V saline oral gavage	[61]
Oral gavage (0.5 mL of 0.139 g whey included 0.188 g leucine) whey protein isolate plus leucine (Lacprodan9224 Arla Foods) Post-prandial study 60, 75, 90 min	25-month-old male C57/BL6J mice *n* = 8	Whey:↑ muscle protein synthesis V fasted or leucine alone ↑ phosphorylated Akt and phosphorylated 4E-BP1 V fasted or leucine alone ↑ phosphorylated p70S6kV fasted	[62]
6 g meal (0.864 g whey) Post prandial study 0, 90, 125, 180, 240 min post ingestion	20-month-old male Wistar rats *n* = 10 per time point	Whey: ↑ muscle protein synthesis (MPS) rate V casein group (diet X time interaction for whey V soy group) ↑ plasma leucine V casein and soy group ↑ phosphorylated Akt, S6K1 and S6: total Akt, S6K1 and S6 S ratio V casein and soy groups with some diet X time interactions	[63]
Whey protein (160 g/kg feed) (Lactalis) 5-month intervention ad libitum and energy-restricted diets	21-month-old male Wistar rats *n* = 10	Ad libitum + whey: Tendency ↑ soleus muscle weight V casein diet Ad libitum + whey and restricted + whey: ↑ muscle protein absolute synthesis rate V casein diet Energy restricted + whey: tendency ↑ muscle strength V casein diet	[64]
Whey protein 0.85 g bolus, 5 days/week over 2 months (+/− exercise)	17-month-old male Wistar rats *n* = 16	Whey: ↑ hind limb stride length of sedentary rats V time zero ↑ maximum voluntary walking speed of active rats V time zero No difference in total movement, distance travelled, activity time or average speed for whey V casein or milk protein intervention ↓ stance time of whey active rats V time zero ↓ brake time of whey active rats V time zero No difference in absolute or relative grip force	[65]
Unilateral hindlimb casting for 8 days followed by 40-day recovery with 144 g/kg feed whey (Prolacta, Lactalis)	22-old-male Wistar rats *n* = 17	Whey: Day20, Day 40 ↑ muscle mass gain V day-1 recovery, control diet and leucine-rich diet Day 40 ↑ muscle protein synthesis V day-1 recovery, control diet and leucine-rich diet	[66]

**Table 3:** Postprandial trials in the older adult with whey intervention. mTOR: mammalian target of rapamycin; FSR: fractional synthesis rate; LEAA: leucine essential amino acid; WP: whey protein; * power calculation to determine number of subjects was performed.

Cohort	Whey Source, Dosage, Duration	Outcome of Whey Supplementation	Reference
*n* = 33 aged 73 ± 2 years, males Randomised	Postprandial L-[1-^13^C] phenylalanine-labeled whey protein 10 g, 20 g, or 35 g containing Givaudan Whey *n* = 11	↓ Whole body protein breakdown V time zero 35 g or 20 g ↑ whole protein synthesis V 10 g whey 35 g ↑ whole protein oxidation V time zero and 10 g whey Dose dependent ↑ whole protein net balance 35 g ↑ muscle fractional protein synthesis rate V 10 g or time zero Dose-dependent ↑ radiolabeled phenylalanine muscle incorporation	[67]
*n* = 27, aged 61 ± 1 years, males Randomised, isocaloric controlled	Postprandial 300 mL whey protein hydrolysate beverage (0.45 g/kg lean body mass) (PEPTIGEN Arla foods) Exercise = leg extensions Whey *n* = 10	↑ Myofilbrillar muscle protein synthesis in rested muscle biopsies V fasted state but similar to casein or maltodextrin control No difference phosphorylated mTOR p70S6K: V casein or maltodextrin in rested or exercised state	[68]
*n* = 20 aged 55–75 years, males Randomised, double blinded, controlled	Postprandial Whey protein isolate 30.4 g (DuPont Nutrition and Health) Exercise = leg extension Whey *n* = 10	↑ Muscle fractional synthesis rates FSR V baseline but no difference to soy-dairy protein blend ↓ Muscle fractional breakdown rate V baseline but no difference to soy-dairy protein blend ↑ mTORC1, S6KI, 4E-BP1, rpS6 V baseline no difference to soy-dairy protein blend	[69]
*n* = 24, aged 65 ± 1 year, females Randomised	Postprandial Whey protein 40 g (Ajinomoto Co.) Exercise = knee extensions Whey *n* = 8	↑ Muscle myofibrillar protein fractional synthesis rate after 2 h for 1.5 g, 6 g LEAA WP and after 4 h for 6 g LEAA + WP V time zero ↑ Muscle myofibrillar protein fractional synthesis rate after 4 h for all t1.5 g, 6 g LEAA and WP + exercise V time zero ↑ Phosphorylated p70S6K1 V time zero ↑ Phosphorylated p70S6K1 for 6 g LEAA and WP + exercise after 2 h and only WP + exercise after 4 h V time zero	[70]
* *n* = 30 healthy (69 ± 1 years old) (*n* = 15) and sarcopenic (81 ± 1 years old) (*n* = 15) males Sarcopenia = gait speed ≤ 1.0 m/s, handgrip strength < 30 kg, SMMI < 8.4 kg/m^2^	Postprandial Leucine enriched whey protein 3 g of leucine/21 g of whey (Nutricia advanced medical nutrition) Whey *n* = 30	↑ Muscle tissue free enrichment, muscle protein bound enrichment gain and mixed muscle protein synthesis rate in both healthy and sarcopenic individuals V time zero	[71]
*n* = 24, ≥60 years, males Randomised, cross-sectional, double blinded, placebo controlled.	Postprandial 0.45 g/kg LBM whey protein isolate containing 21.3–37.6 g protein (Lacprodan, Arla Foods) Exercise = knee extension Whey *n* = 14	↑ Myofibrillar FSR in resting and exercised legs V time zero ↑ Connective tissue FSR for whey + exercise V time zero Tendency towards a difference between postprandial & post exercise states for both whey and whey + ibuprofen groups	[72]
*n* = 22 aged 50 to 65 years postmenopausal women Randomised	Postprandial Whey protein trial consumed either 0.6 g whey protein per kg FFM (ProSynthesis Laboratories) Whey *n* = 11	↑ Phosphorylated mTOR^Ser2448^& p70S6K^Thr389^ in muscle V time zero and control values (Kool Aid solution only) ↑ Phosphorylated AKT^Ser473^ and AKT^Thr308^ in muscle V time zeros	[73]

**Table 4:** Whey and exercise intervention trials with the older adult. SOD: superoxide dismutase; CAT: catalase; TRAP: total radical-trapping antioxidant parameter; AOPP: advanced oxidation protein products; LST: lean soft tissue; CSA: cross-sectional area; IL-6: interleukin 6; WPS: whey protein supplementation; SPPB: Short Physical Performance Battery; FRAP: ferric reducing antioxidant power; GSH: glutathione; * power calculation to determine number of subjects was performed.

Cohort	Whey Source, Dosage, Duration	Outcome of Whey Supplementation	Reference
*n* = 70 ≥ 60 years, females Randomised, double-blind, placebo-controlled	26 weeks 35 g hydrolyzed whey protein (Lacprodan, Arla Foods) Exercise = chest press, horizontal leg press, seated row, knee extension, preacher curl, leg curl, triceps pushdown, and seated calf raise Whey *n* = 47	↓ Uric acid V maltodextrin. ↑ SOD, CAT and TRAP V time zero ↓ AOPP and lipid hydroperoxide V time zero	[76]
*n* = 70 ≥ 60 year, females Randomised, double-blind, placebo-controlled	26 weeks 35 g hydrolyzed whey protein (Lacprodan, Arla Foods) Exercise = chest press, knee extension & preacher curl Whey *n* = 47	↑ Total lean soft tissue, appendicular LST, lower LST & V time zero and maltodextrin ↓ 10-min walk speed and rising from seated position V time zero equal to maltodextrin ↑ Knee extension, preachers curls, chest press and total strength V time zero equal to maltodextrin	[75]
*n* = 31, 67.4 ± 4.0 years, females 8-week pre-conditioned resistance training Randomised, double-blind controlled	12 weeks 35 g hydrolysed whey protein (Lacprodan, Arla Foods) Exercise = chest press, knee extension and preacher curl Whey *n* = 13	↑ Chest press, knee extension, preacher curl, total strength, lean soft tissue, muscle mass, muscle quality index V 8-week exercise alone ↑ Chest press, knee extension total strength V maltodextrin with exercise	[77]
* *n* = 75, aged 65–80 years, females Randomised, single-blind controlled.	24 weeks 22.3 g whey protein (Ezaki Glico) Exercise = rising and sitting from a chair, and leg extension with resistance band exercises Whey *n* = 54	Exercise + whey > exercise > whey for ↑ limb muscle mass, skeletal muscle mass index, grip strength, knee extension, gait speed	[78]
* *n* = 149 aged 78.5 ± 5.4 years, males and females Randomised, double blinded, placebo-controlled	6-month period whey protein 20 g + Vitamin D 800 IU (Nestle Health Science) Exercise = 30 min aerobic + 20 min strength Whey *n* = 74	↑ Normal density muscle V controls (a low-calorie placebo drink -30 kcal, no protein, no Vitamin D) and time zero ↓ Low-density muscle V time zero ↓ Intermuscular fat V controls and time zero ↑ Knee flexor strength, power and quality for both supplemented and control groups V baseline values	[79,80]
* *n* = 80 aged 70–85 years, males and females Randomised, double-blind, controlled	6 months Whey protein concentrate 40 g/daily (Innovative food processors Inc.) Exercise = walking or stationary cycling, knee extension and leg press Whey *n* = 42	↑ Knee extensor power, total lean mass, total mid-thigh CSA, total muscle CSA and total normal density muscle CSA V maltodextrin ↑ Peak power for double leg press V time zero, but similar to maltodextrin ↓ Stair climb and chair rise time V time zero	[81]
* *n* = 46 aged ≥60 – 86 years, males and females Randomised, single-blind	16 weeks Leucine (0.03 g/kg/meal) enriched whey protein (0.5 g/kg/day) (MyProtein) Exercise = leg press, chest press and bicep curl Whey *n* = 22	↑ Leg press, chest press and bicep curl V time zero, no differences between whey + exercise or exercise groups ↑ SPPB score V time zero	[82]
* *n* = 100 aged 69 ± 6 years, males and females Randomised, single-blind, controlled	16 weeks Leucine (0.03 g/kg/meal) enriched whey protein (0.5 g/kg/day) (MyProtein) Exercise = leg press, chest press, calf press, shoulder press, seated row, back extension and bicep curl Whey *n* = 45	Rectus femoris and bicep femoris were more resistant to fatigue for both exercise intervention V time zero No additional benefit with supplementation of whey	[83]
*n* = 65, aged = 60–93 years, hospitalised males and females Single-blind test, controlled	Length of hospital stay 24 g of whey protein Rehabilitation programme Whey *n* = 27	↑ Grip strength, knee extensor force V time zero improved gait speed, and timed up and go V time zero ↓ IL-6 V time zero for both WPS group & control group (hot cereal and milk products - WPS) ˃↓ in % change of IL-6 V controls (hot cereal and milk products - WPS)	[84]
* *n* = 165 aged >70 years, males and females hospitalised subjects Block randomised, double-blinded, placebo-controlled	Length of hospitalization and 12 weeks after discharge A protein-enriched, milk-based supplement beverage–26.25 g whey protein (Protino, Arla foods) Exercise = bridging exercise, rising from a chair, and heel-raise Whey *n* = 83	No noted benefit of whey protein supplementation Iso-energetic placebo-products (<1.5 g protein) = improved 30 sec chair stand test, 4 m gait speed, ↑ handgrip strength V time zero	[85,86]
*n* = 69 aged 52.5 ± 15.2 years, males and females. Double-blind, cross-over study	30 days Whey based oral supplement-10 g/day (ProtherSOD) Exercise = cycle ergometer Whey *n* = 13	↑ FRAP and GSH levels V time zero and casein ↓ AOPP V time zero and casein	[87]

**Table 5:** Older adult intervention with whey protein supplementation but no exercise. IGF-1: Insulin growth factor 1; EAAMR: Essential amino acid meal replacement; FSR: fractional synthesis rate. * power calculation to determine number of subjects was performed.

Cohort	Whey Source, Dosage, Duration	Outcome of Whey Supplementation	Reference
* *n* = 24 aged 71 ± 4 years, males Randomised, double-blind, placebo-controlled	6 weeks Leucine (3 g) enriched whey protein (20 g) in combination with vitamin D (800 IU) (Nutricia Advanced Medical Nutrition) Whey *n* = 12	↑ Mixed muscle protein synthesis rate V time zero and control (flavored water drink). Controls = ↑ V time zero ↑ Appendicular muscle mass and leg lean mass V flavored water	[88]
*n* = 52 aged 85.0 ± 7.4 years, males and females Randomised, double-blind, controlled	28-day intervention 15 g whey protein + 5 g essential amino acids + 550 mg Ca (Novartis Nutrition) Whey *n* = 61	↑ Activity of daily living score for whey ± zinc V time zero ↑ IGF-1 in whey ± zinc V time zero	[89,90]
*n* = 12 aged 65–80 years, males and females Randomised	8 weeks EAAMR: Essential amino acid meal replacement–7 g intact whey protein Whey *n* = 6	↑ Skeletal muscle FSR V control group (competitive meal replacer)	[91]

**Table 6:** Whey intervention trials involving older individuals with sarcopenia. LST: Lean soft tissue; IL-6: Interleukin 6; TNF-α: Tumor necrosis factor alpha; CRP: C reactive protein; BIA: Bio-Impedance Analysis; IL-1Ra: Interleukin 1 receptor antagonist; IL-8: Interleukin 8; RSMI: Relative muscle mass index; IGF-1: Insulin-like growth factor 1; IL-2: Interleukin 2; TWEAK—Tumor necrosis factor-like WEAK inducer of apoptosis; IL-18 Interleukin 18; SPPB: Short Physical Performance Battery. * power calculation to determine number of subjects was performed.

Cohort	Whey Source, Dosage, Duration	Outcome of Whey Supplementation	Reference
*n* = 26 ≥ 60 years, females sarcopenic obesity—fat mass ≥35% & appendicular lean soft tissue ≤15.02 kg Randomised, double blind, placebo controlled	16 weeks 35 g hydrolysed whey protein (Lacprodan, Arla Foods) Exercise = chest press, horizontal leg press, seated row, knee extension, preacher curl, leg curl, triceps pushdown, and seated calf raise Whey *n* = 13	↑ LST V maltodextrin and time zero ↓ Total LST, relative total fat mass and trunk fat mass V maltodextrin ↓ Number of sarcopenia and sarcopenic obesity V time zero ↓ Blood IL-6 V time zero and maltodextrin ↑ Knee extension, chest press, preacher curl, total strength V time zero but no difference to maltodextrin ↓ Blood TNF-a, CRP, advanced oxidation products V time zero but not maltodextrin ↑ Blood total radical trapping antioxidant potential V time zero but not maltodextrin	[92]
* *n* = 297 aged ≥ 65 years, males and females Sarcopenic subjects: BIA < 37% in men and <28% women Randomised double blind isocaloric controlled.	13 weeks 20 g whey protein, 3 g total leucine, and 800 IU vitamin D Whey *n* = 137	↑ IL-6, IL-1Ra & CRP for control (31.4 g of carbohydrates) and supplemented groups V time zero ↓ IL-8 V time zero	[93]
*n* = 60 aged 60–85 years, males and females Sarcopenic subject: RSMI < 5.7 kg/m^2^ for women and <7.0 kg/m^2^ for men Double blind randomized controlled	6-month 22 g whey protein plus vitamin D (702 IU) & E (109 mg) per serving Whey *n* = 30	↑ Appendicular muscle mass, RSMI, handgrip strength, IGF-1 V isocaloric placebo (32.4 g of carbohydrates, 2.6 g of fat) ↓ IL-2 V isocaloric placebo No difference TNF-a, IL-6, CRP V isocaloric placebo or time zero ↓ Time to stand V isocaloric placebo	[94]
* *n* = 112 males & females, n = 56 sarcopenic aged 72.05 ± 6.54, n = 56 non sarcopenic aged 65.24 ± 4.05 Sarcopenia subjects: RSMI 7.0 kg/m^2^ men, 5.4 kg/m^2^ women	12 weeks 30 g of whey with 3.84 g total leucine. (Lacprodan, Arla Foods) Exercise = 5 min warm-up, 20 min muscle strength training, and 5 min slow walking	Sarcopenic group: ↑ muscle mass V time zero ↓ TWERK, TNF-α and IL-18 V time zero. ↑ IGF-1 V time zero	[95]
* *n* = 380 aged > 65 years, males & females Sarcopenia = SPPB score between 4–9 and a skeletal muscle index of ≤37% in men and ≤28% in women Randomised, double-blind, controlled	13 weeks Leucine (3 g) enriched whey protein (20 g) Whey *n* = 184	↑ IGF-1, V time zero.↓ in IGF-1 for control group (isocaloric product) V time zero ↓ Chair stand test time and gait speed V time zero for both test and control groups ↑ SPPB score V time zero for both test and isocaloric product	[96] [97]

## References

[1] Tieland M., Trouwborst I., Clark B.C. (2018). Skeletal muscle performance and ageing. J. Cachexiasarcopenia Muscle.

[2] Aagaard P., Suetta C., Caserotti P., Magnusson S.P., Kjaer M. (2010). Role of the nervous system in sarcopenia and muscle atrophy with aging: Strength training as a countermeasure. Scand. J. Med. Sci. Sports.

[3] Kalyani R.R., Corriere M., Ferrucci L. (2014). Age-related and disease-related muscle loss: The effect of diabetes, obesity, and other diseases. Lancet Diabetes Endocrinol..

[4] Patel H.P., Syddall H.E., Jameson K., Robinson S., Denison H., Roberts H.C., Edwards M., Dennison E., Cooper C., Aihie Sayer A. (2013). Prevalence of sarcopenia in community-dwelling older people in the UK using the European Working Group on Sarcopenia in Older People (EWGSOP) definition: Findings from the Hertfordshire Cohort Study (HCS). Age Ageing.

[5] MacEwan J.P., Gill T.M., Johnson K., Doctor J., Sullivan J., Shim J., Goldman D.P. (2018). Measuring Sarcopenia Severity in Older Adults and the Value of Effective Interventions. J. Nutr. Health Aging.

[6] Von Haehling S., Morley J.E., Anker S.D. (2010). An overview of sarcopenia: Facts and numbers on prevalence and clinical impact. J. Cachexiasarcopenia Muscle.

[7] Ethgen O., Beaudart C., Buckinx F., Bruyere O., Reginster J.Y. (2017). The Future Prevalence of Sarcopenia in Europe: A Claim for Public Health Action. Calcif. Tissue Int..

[8] Shafiee G., Keshtkar A., Soltani A., Ahadi Z., Larijani B., Heshmat R. (2017). Prevalence of sarcopenia in the world: A systematic review and meta- analysis of general population studies. J. Diabetes Metab. Disord..

[9] Cruz-Jentoft A.J., Bahat G., Bauer J., Boirie Y., Bruyère O., Cederholm T., Cooper C., Landi F., Rolland Y., Sayer A.A. (2019). Sarcopenia: Revised European consensus on definition and diagnosis. Age Ageing.

[10] Chumlea W.C., Cesari M., Evans W.J., Ferrucci L., Fielding R.A., Pahor M., Studenski S., Vellas B., The Task Force M. (2011). International working group on Sarcopenia. J. Nutr. Health Aging.

[11] Chen L.K., Woo J., Assantachai P., Auyeung T.W., Chou M.Y., Iijima K., Jang H.C., Kang L., Kim M., Kim S. (2020). Asian Working Group for Sarcopenia: 2019 Consensus Update on Sarcopenia Diagnosis and Treatment. J. Am. Med. Dir. Assoc..

[12] Messina C., Maffi G., Vitale J.A., Ulivieri F.M., Guglielmi G., Sconfienza L.M. (2018). Diagnostic imaging of osteoporosis and sarcopenia: A narrative review. Quant. Imaging Med. Surg..

[13] Kim J., Wang Z., Heymsfield S.B., Baumgartner R.N., Gallagher D. (2002). Total-body skeletal muscle mass: Estimation by a new dual-energy X-ray absorptiometry method. Am. J. Clin. Nutr..

[14] Guimarães-Ferreira L. (2014). Role of the phosphocreatine system on energetic homeostasis in skeletal and cardiac muscles. Einstein (Sao Paulo, Brazil).

[15] Stevens L.A., Coresh J., Greene T., Levey A.S. (2006). Assessing kidney function--measured and estimated glomerular filtration rate. N. Engl. J. Med..

[16] Patel S.S., Molnar M.Z., Tayek J.A., Ix J.H., Noori N., Benner D., Heymsfield S., Kopple J.D., Kovesdy C.P., Kalantar-Zadeh K. (2013). Serum creatinine as a marker of muscle mass in chronic kidney disease: Results of a cross-sectional study and review of literature. J. Cachexiasarcopenia Muscle.

[17] Rong Y.-D., Bian A.-L., Hu H.-Y., Ma Y., Zhou X.-Z. (2018). Study on relationship between elderly sarcopenia and inflammatory cytokine IL-6, anti-inflammatory cytokine IL-10. BMC Geriatr..

[18] Bian A.-L., Hu H.-Y., Rong Y.-D., Wang J., Wang J.-X., Zhou X.-Z. (2017). A study on relationship between elderly sarcopenia and inflammatory factors IL-6 and TNF-α. Eur. J. Med Res..

[19] Osaka T., Hamaguchi M., Hashimoto Y., Ushigome E., Tanaka M., Yamazaki M., Fukui M. (2018). Decreased the creatinine to cystatin C ratio is a surrogate marker of sarcopenia in patients with type 2 diabetes. Diabetes Res. Clin. Pr..

[20] Ferrucci L., Fabbri E. (2018). Inflammageing: Chronic inflammation in ageing, cardiovascular disease, and frailty. Nat. Rev. Cardiol..

[21] Kwak J.Y., Hwang H., Kim S.-K., Choi J.Y., Lee S.-M., Bang H., Kwon E.-S., Lee K.-P., Chung S.G., Kwon K.-S. (2018). Prediction of sarcopenia using a combination of multiple serum biomarkers. Sci. Rep..

[22] Taaffe D.R., Harris T.B., Ferrucci L., Rowe J., Seeman T.E. (2000). Cross-sectional and prospective relationships of interleukin-6 and C-reactive protein with physical performance in elderly persons: MacArthur studies of successful aging. J. Gerontol. Ser. Abiological Sci. Med Sci..

[23] Bano G., Trevisan C., Carraro S., Solmi M., Luchini C., Stubbs B., Manzato E., Sergi G., Veronese N. (2017). Inflammation and sarcopenia: A systematic review and meta-analysis. Maturitas.

[24] Stowe R.P., Peek M.K., Goodwin J.S., Cutchin M.P. (2009). Plasma Cytokine Levels in a Population-Based Study: Relation to Age and Ethnicity. J. Gerontol. Ser. A.

[25] Leger B., Derave W., De Bock K., Hespel P., Russell A.P. (2008). Human sarcopenia reveals an increase in SOCS-3 and myostatin and a reduced efficiency of Akt phosphorylation. Rejuvenation Res..

[26] Barclay R.D., Burd N.A., Tyler C., Tillin N.A., MacKenzie R.W. (2019). The Role of the IGF-1 Signaling Cascade in Muscle Protein Synthesis and Anabolic Resistance in Aging Skeletal Muscle. Front. Nutr..

[27] Ashpole N.M., Herron J.C., Estep P.N., Logan S., Hodges E.L., Yabluchanskiy A., Humphrey M.B., Sonntag W.E. (2016). Differential effects of IGF-1 deficiency during the life span on structural and biomechanical properties in the tibia of aged mice. Age (Dordr).

[28] Ng T.P., Lu Y., Choo R.W.M., Tan C.T.Y., Nyunt M.S.Z., Gao Q., Mok E.W.H., Larbi A. (2018). Dysregulated homeostatic pathways in sarcopenia among frail older adults. Aging Cell.

[29] Pansarasa O., Castagna L., Colombi B., Vecchiet J., Felzani G., Marzatico F. (2000). Age and sex differences in human skeletal muscle: Role of reactive oxygen species. Free Radic. Res..

[30] Young I.S., Woodside J.V. (2001). Antioxidants in health and disease. J. Clin. Pathol..

[31] Powers S.K., Ji L.L., Kavazis A.N., Jackson M.J. (2011). Reactive oxygen species: Impact on skeletal muscle. Compr. Physiol..

[32] Powers S.K., Jackson M.J. (2008). Exercise-induced oxidative stress: Cellular mechanisms and impact on muscle force production. Physiol. Rev..

[33] Kurutas E.B. (2016). The importance of antioxidants which play the role in cellular response against oxidative/nitrosative stress: Current state. Nutr. J..

[34] Liguori I., Russo G., Curcio F., Bulli G., Aran L., Della-Morte D., Gargiulo G., Testa G., Cacciatore F., Bonaduce D. (2018). Oxidative stress, aging, and diseases. Clin. Interv. Aging.

[35] Frontera W.R., Ochala J. (2015). Skeletal muscle: A brief review of structure and function. Calcif. Tissue Int..

[36] Liao Y., Peng Z., Chen L., Zhang Y., Cheng Q., Nüssler A.K., Bao W., Liu L., Yang W. (2019). Prospective Views for Whey Protein and/or Resistance Training Against Age-related Sarcopenia. Aging Dis.

[37] West D.W.D., Abou Sawan S., Mazzulla M., Williamson E., Moore D.R. (2017). Whey Protein Supplementation Enhances Whole Body Protein Metabolism and Performance Recovery after Resistance Exercise: A Double-Blind Crossover Study. Nutrients.

[38] Davies R.W., Carson B.P., Jakeman P.M. (2018). The Effect of Whey Protein Supplementation on the Temporal Recovery of Muscle Function Following Resistance Training: A Systematic Review and Meta-Analysis. Nutrients.

[39] Stark M., Lukaszuk J., Prawitz A., Salacinski A. (2012). Protein timing and its effects on muscular hypertrophy and strength in individuals engaged in weight-training. J. Int. Soc. Sports Nutr..

[40] Rutherfurd S.M., Fanning A.C., Miller B.J., Moughan P.J. (2014). Protein Digestibility-Corrected Amino Acid Scores and Digestible Indispensable Amino Acid Scores Differentially Describe Protein Quality in Growing Male Rats. J. Nutr..

[41] Brennan J.L., Keerati-U-Rai M., Yin H., Daoust J., Nonnotte E., Quinquis L., St-Denis T., Bolster D.R. (2019). Differential Responses of Blood Essential Amino Acid Levels Following Ingestion of High-Quality Plant-Based Protein Blends Compared to Whey Protein-A Double-Blind Randomized, Cross-Over, Clinical Trial. Nutrients.

[42] Almeida C.C., Alvares T.S., Costa M.P., Conte-Junior C.A. (2016). Protein and Amino Acid Profiles of Different Whey Protein Supplements. J. Diet. Suppl..

[43] Hulmi J.J., Lockwood C.M., Stout J.R. (2010). Effect of protein/essential amino acids and resistance training on skeletal muscle hypertrophy: A case for whey protein. Nutr. Metab. (Lond.).

[44] Tosukhowong P., Boonla C., Dissayabutra T., Kaewwilai L., Muensri S., Chotipanich C., Joutsa J., Rinne J., Bhidayasiri R. (2016). Biochemical and clinical effects of Whey protein supplementation in Parkinson’s disease: A pilot study. J. Neurol. Sci..

[45] Hulmi J.J., Tannerstedt J., Selanne H., Kainulainen H., Kovanen V., Mero A.A. (2009). Resistance exercise with whey protein ingestion affects mTOR signaling pathway and myostatin in men. J. Appl. Physiol. (Bethesda Md. 1985).

[46] Brandelli A., Daroit D.J., Corrêa A.P.F. (2015). Whey as a source of peptides with remarkable biological activities. Food Res. Int..

[47] Madureira A.R., Pereira C.I., Gomes A.M.P., Pintado M.E., Xavier Malcata F. (2007). Bovine whey proteins–Overview on their main biological properties. Food Res. Int..

[48] Adams R.L., Broughton K.S. (2016). Insulinotropic Effects of Whey: Mechanisms of Action, Recent Clinical Trials, and Clinical Applications. Ann. Nutr. Metab..

[49] Corrochano A.R., Ferraretto A., Arranz E., Stuknyte M., Bottani M., O’Connor P.M., Kelly P.M., De Noni I., Buckin V., Giblin L. (2019). Bovine whey peptides transit the intestinal barrier to reduce oxidative stress in muscle cells. Food Chem..

[50] Ogiwara M., Ota W., Mizushige T., Kanamoto R., Ohinata K. (2018). Enzymatic digest of whey protein and wheylin-1, a dipeptide released in the digest, increase insulin sensitivity in an Akt phosphorylation-dependent manner. Food Funct..

[51] Mobley C.B., Fox C.D., Thompson R.M., Healy J.C., Santucci V., Kephart W.C., McCloskey A.E., Kim M., Pascoe D.D., Martin J.S. (2016). Comparative effects of whey protein versus l-leucine on skeletal muscle protein synthesis and markers of ribosome biogenesis following resistance exercise. Amino Acids.

[52] Frey J.W., Jacobs B.L., Goodman C.A., Hornberger T.A. (2014). A role for Raptor phosphorylation in the mechanical activation of mTOR signaling. Cell Signal.

[53] Kerasioti E., Stagos D., Priftis A., Aivazidis S., Tsatsakis A.M., Hayes A.W., Kouretas D. (2014). Antioxidant effects of whey protein on muscle C2C12 cells. Food Chem..

[54] Xu R., Liu N., Xu X., Kong B. (2011). Antioxidative effects of whey protein on peroxide-induced cytotoxicity. J. Dairy Sci..

[55] Knight M.I., Tester A.M., McDonagh M.B., Brown A., Cottrell J., Wang J., Hobman P., Cocks B.G. (2014). Milk-derived ribonuclease 5 preparations induce myogenic differentiation in vitro and muscle growth in vivo. J. Dairy Sci..

[56] Carson B.P., Patel B., Amigo-Benavent M., Pauk M., Kumar Gujulla S., Murphy S.M., Kiely P.A., Jakeman P.M. (2018). Regulation of muscle protein synthesis in an in vitro cell model using ex vivo human serum. Exp. Physiol..

[57] Dutta S., Sengupta P. (2016). Men and mice: Relating their ages. Life Sci..

[58] Andreollo N.A., Santos E.F.d., Araújo M.R., Lopes L.R. (2012). Idade dos ratos versus idade humana: Qual é a relação?. Abcd. Arq. Bras. De Cir. Dig. (São Paulo).

[59] Van Dijk M., Dijk F.J., Bunschoten A., van Dartel D.A., van Norren K., Walrand S., Jourdan M., Verlaan S., Luiking Y. (2016). Improved muscle function and quality after diet intervention with leucine-enriched whey and antioxidants in antioxidant deficient aged mice. Oncotarget.

[60] Mosoni L., Gatineau E., Gatellier P., Migne C., Savary-Auzeloux I., Remond D., Rocher E., Dardevet D. (2014). High whey protein intake delayed the loss of lean body mass in healthy old rats, whereas protein type and polyphenol/antioxidant supplementation had no effects. PLoS ONE.

[61] Garg G., Singh S., Kumar Singh A., Ibrahim Rizvi S. (2018). Whey protein concentrate supplementation protects erythrocyte membrane from aging-induced alterations in rats. J. Food Biochem..

[62] Dijk F.J., van Dijk M., Walrand S., van Loon L.J.C., van Norren K., Luiking Y.C. (2018). Differential effects of leucine and leucine-enriched whey protein on skeletal muscle protein synthesis in aged mice. Clin. Nutr. ESPEN.

[63] Jarzaguet M., Polakof S., David J., Migne C., Joubrel G., Efstathiou T., Remond D., Mosoni L., Dardevet D. (2018). A meal with mixed soy/whey proteins is as efficient as a whey meal in counteracting the age-related muscle anabolic resistance only if the protein content and leucine levels are increased. Food Funct..

[64] Walrand S., Zangarelli A., Guillet C., Salles J., Soulier K., Giraudet C., Patrac V., Boirie Y. (2011). Effect of fast dietary proteins on muscle protein synthesis rate and muscle strength in ad libitum-fed and energy-restricted old rats. Br. J. Nutr..

[65] Lafoux A., Baudry C., Bonhomme C., Le Ruyet P., Huchet C. (2016). Soluble Milk Protein Supplementation with Moderate Physical Activity Improves Locomotion Function in Aging Rats. PLoS ONE.

[66] Magne H., Savary-Auzeloux I., Migné C., Peyron M.-A., Combaret L., Rémond D., Dardevet D. (2012). Contrarily to whey and high protein diets, dietary free leucine supplementation cannot reverse the lack of recovery of muscle mass after prolonged immobilization during ageing. J. Physiol..

[67] Pennings B., Groen B., Lange A.d., Gijsen A.P., Zorenc A.H., Senden J.M.G., Loon L.J.C.v. (2012). Amino acid absorption and subsequent muscle protein accretion following graded intakes of whey protein in elderly men. Am. J. Physiol. Endocrinol. Metab..

[68] Reitelseder S., Dideriksen K., Agergaard J., Malmgaard-Clausen N.M., Bechshoeft R.L., Petersen R.K., Serena A., Mikkelsen U.R., Holm L. (2019). Even effect of milk protein and carbohydrate intake but no further effect of heavy resistance exercise on myofibrillar protein synthesis in older men. Eur. J. Nutr..

[69] Borack M.S., Reidy P.T., Husaini S.H., Markofski M.M., Deer R.R., Richison A.B., Lambert B.S., Cope M.B., Mukherjea R., Jennings K. (2016). Soy-Dairy Protein Blend or Whey Protein Isolate Ingestion Induces Similar Postexercise Muscle Mechanistic Target of Rapamycin Complex 1 Signaling and Protein Synthesis Responses in Older Men. J. Nutr..

[70] Wilkinson D.J., Bukhari S.S.I., Phillips B.E., Limb M.C., Cegielski J., Brook M.S., Rankin D., Mitchell W.K., Kobayashi H., Williams J.P. (2018). Effects of leucine-enriched essential amino acid and whey protein bolus dosing upon skeletal muscle protein synthesis at rest and after exercise in older women. Clin. Nutr. (Edinb. Scotl.).

[71] Kramer I.F., Verdijk L.B., Hamer H.M., Verlaan S., Luiking Y.C., Kouw I.W.K., Senden J.M., van Kranenburg J., Gijsen A.P., Bierau J. (2017). Both basal and post-prandial muscle protein synthesis rates, following the ingestion of a leucine-enriched whey protein supplement, are not impaired in sarcopenic older males. Clin. Nutr. (Edinb. Scotl.).

[72] Dideriksen K., Reitelseder S., Malmgaard-Clausen N.M., Bechshoeft R., Petersen R.K., Mikkelsen U.R., Holm L. (2016). No effect of anti-inflammatory medication on postprandial and postexercise muscle protein synthesis in elderly men with slightly elevated systemic inflammation. Exp. Gerontol..

[73] Smith G.I., Yoshino J., Stromsdorfer K.L., Klein S.J., Magkos F., Reeds D.N., Klein S., Mittendorfer B. (2015). Protein Ingestion Induces Muscle Insulin Resistance Independent of Leucine-Mediated mTOR Activation. Diabetes.

[74] Mayer F., Scharhag-Rosenberger F., Carlsohn A., Cassel M., Müller S., Scharhag J. (2011). The intensity and effects of strength training in the elderly. Dtsch. Arztebl. Int..

[75] Nabuco H.C.G., Tomeleri C.M., Sugihara Junior P., Fernandes R.R., Cavalcante E.F., Antunes M., Ribeiro A.S., Teixeira D.C., Silva A.M., Sardinha L.B. (2018). Effects of Whey Protein Supplementation Pre- or Post-Resistance Training on Muscle Mass, Muscular Strength, and Functional Capacity in Pre-Conditioned Older Women: A Randomized Clinical Trial. Nutrients.

[76] Nabuco H.C.G., Tomeleri C.M., Fernandes R.R., Sugihara Junior P., Venturini D., Barbosa D.S., Deminice R., Sardinha L.B., Cyrino E.S. (2019). Effects of pre- or post-exercise whey protein supplementation on oxidative stress and antioxidant enzymes in older women. Scand. J. Med. Sci. Sports.

[77] Sugihara Junior P., Ribeiro A.S., Nabuco H.C.G., Fernandes R.R., Tomeleri C.M., Cunha P.M., Venturini D., Barbosa D.S., Schoenfeld B.J., Cyrino E.S. (2018). Effects of Whey Protein Supplementation Associated With Resistance Training on Muscular Strength, Hypertrophy, and Muscle Quality in Preconditioned Older Women. Int. J. Sport Nutr. Exerc. Metab..

[78] Mori H., Tokuda Y. (2018). Effect of whey protein supplementation after resistance exercise on the muscle mass and physical function of healthy older women: A randomized controlled trial. Geriatr. Gerontol. Int..

[79] Englund D.A., Kirn D.R., Koochek A., Zhu H., Travison T.G., Reid K.F., von Berens A., Melin M., Cederholm T., Gustafsson T. (2017). Nutritional Supplementation With Physical Activity Improves Muscle Composition in Mobility-Limited Older Adults, The VIVE2 Study: A Randomized, Double-Blind, Placebo-Controlled Trial. J. Gerontol. Ser. Abiological Sci. Med Sci..

[80] Fielding R.A., Travison T.G., Kirn D.R., Koochek A., Reid K.F., von Berens A., Zhu H., Folta S.C., Sacheck J.M., Nelson M.E. (2017). Effect of Structured Physical Activity and Nutritional Supplementation on Physical Function in Mobility-Limited Older Adults: Results from the VIVE2 Randomized Trial. J. Nutr. Health Aging.

[81] Chalé A., Cloutier G.J., Hau C., Phillips E.M., Dallal G.E., Fielding R.A. (2013). Efficacy of whey protein supplementation on resistance exercise-induced changes in lean mass, muscle strength, and physical function in mobility-limited older adults. J. Gerontol. Ser. Abiological Sci. Med Sci..

[82] Kirk B., Mooney K., Amirabdollahian F., Khaiyat O. (2019). Exercise and Dietary-Protein as a Countermeasure to Skeletal Muscle Weakness: Liverpool Hope University–Sarcopenia Aging Trial (LHU-SAT). Front. Physiol..

[83] Kirk B., Mooney K., Cousins R., Angell P., Jackson M., Pugh J.N., Coyles G., Amirabdollahian F., Khaiyat O. (2020). Effects of exercise and whey protein on muscle mass, fat mass, myoelectrical muscle fatigue and health-related quality of life in older adults: A secondary analysis of the Liverpool Hope University-Sarcopenia Ageing Trial (LHU-SAT). Eur. J. Appl. Physiol..

[84] Niccoli S., Kolobov A., Bon T., Rafilovich S., Munro H., Tanner K., Pearson T., Lees S.J. (2017). Whey Protein Supplementation Improves Rehabilitation Outcomes in Hospitalized Geriatric Patients: A Double Blinded, Randomized Controlled Trial. J. Nutr. Gerontol. Geriatr..

[85] Gade J., Beck A.M., Bitz C., Christensen B., Klausen T.W., Vinther A., Astrup A. (2018). Protein-enriched, milk-based supplement to counteract sarcopenia in acutely ill geriatric patients offered resistance exercise training during and after hospitalisation: Study protocol for a randomised, double-blind, multicentre trial. BMJ Open.

[86] Gade J., Beck A.M., Andersen H.E., Christensen B., Ronholt F., Klausen T.W., Vinther A., Astrup A. (2019). Protein supplementation combined with low-intensity resistance training in geriatric medical patients during and after hospitalization: A randomized, double-blind, multicenter trial. Br. J. Nutr..

[87] Mancuso M., Orsucci D., Logerfo A., Rocchi A., Petrozzi L., Nesti C., Galetta F., Santoro G., Murri L., Siciliano G. (2010). Oxidative stress biomarkers in mitochondrial myopathies, basally and after cysteine donor supplementation. J. Neurol..

[88] Chanet A., Verlaan S., Salles J., Giraudet C., Patrac V., Pidou V., Pouyet C., Hafnaoui N., Blot A., Cano N. (2017). Supplementing Breakfast with a Vitamin D and Leucine–Enriched Whey Protein Medical Nutrition Drink Enhances Postprandial Muscle Protein Synthesis and Muscle Mass in Healthy Older Men. J. Nutr..

[89] Ascenzi F., Barberi L., Dobrowolny G., Villa Nova Bacurau A., Nicoletti C., Rizzuto E., Rosenthal N., Scicchitano B.M., Musarò A. (2019). Effects of IGF-1 isoforms on muscle growth and sarcopenia. Aging Cell.

[90] Rodondi A., Ammann P., Ghilardi-Beuret S., Rizzoli R. (2009). Zinc increases the effects of essential amino acids-whey protein supplements in frail elderly. J. Nutr. Health Aging.

[91] Coker R.H., Miller S., Schutzler S., Deutz N., Wolfe R.R. (2012). Whey protein and essential amino acids promote the reduction of adipose tissue and increased muscle protein synthesis during caloric restriction-induced weight loss in elderly, obese individuals. Nutr. J..

[92] Nabuco H.C.G., Tomeleri C.M., Fernandes R.R., Sugihara Junior P., Cavalcante E.F., Cunha P.M., Antunes M., Nunes J.P., Venturini D., Barbosa D.S. (2019). Effect of whey protein supplementation combined with resistance training on body composition, muscular strength, functional capacity, and plasma-metabolism biomarkers in older women with sarcopenic obesity: A randomized, double-blind, placebo-controlled trial. Clin. Nutr. ESPEN.

[93] Liberman K., Njemini R., Luiking Y., Forti L.N., Verlaan S., Bauer J.M., Memelink R., Brandt K., Donini L.M., Maggio M. (2019). Thirteen weeks of supplementation of vitamin D and leucine-enriched whey protein nutritional supplement attenuates chronic low-grade inflammation in sarcopenic older adults: The PROVIDE study. Aging Clin. Exp. Res..

[94] Bo Y., Liu C., Ji Z., Yang R., An Q., Zhang X., You J., Duan D., Sun Y., Zhu Y. (2019). A high whey protein, vitamin D and E supplement preserves muscle mass, strength, and quality of life in sarcopenic older adults: A double-blind randomized controlled trial. Clin. Nutr..

[95] Li C.-W., Yu K., Shyh-Chang N., Li G.-X., Jiang L.-J., Yu S.-L., Xu L.-Y., Liu R.-J., Guo Z.-J., Xie H.-Y. (2019). Circulating factors associated with sarcopenia during ageing and after intensive lifestyle intervention. J. Cachexiasarcopenia Muscle.

[96] Hill T.R., Verlaan S., Biesheuvel E., Eastell R., Bauer J.M., Bautmans I., Brandt K., Donini L.M., Maggio M., Mets T. (2019). A Vitamin D, Calcium and Leucine-Enriched Whey Protein Nutritional Supplement Improves Measures of Bone Health in Sarcopenic Non-Malnourished Older Adults: The PROVIDE Study. Calcif. Tissue Int..

[97] Bauer J.M., Verlaan S., Bautmans I., Brandt K., Donini L.M., Maggio M., McMurdo M.E., Mets T., Seal C., Wijers S.L. (2015). Effects of a vitamin D and leucine-enriched whey protein nutritional supplement on measures of sarcopenia in older adults, the PROVIDE study: A randomized, double-blind, placebo-controlled trial. J. Am. Med. Dir. Assoc..

[98] Il’yasova D., Scarbrough P., Spasojevic I. (2012). Urinary biomarkers of oxidative status. Clin. Chim. Acta.

[99] Cornish S.M., Candow D.G., Jantz N.T., Chilibeck P.D., Little J.P., Forbes S., Abeysekara S., Zello G.A. (2009). Conjugated linoleic acid combined with creatine monohydrate and whey protein supplementation during strength training. Int. J. Sport Nutr. Exerc. Metab..

[100] Marrocco I., Altieri F., Peluso I. (2017). Measurement and Clinical Significance of Biomarkers of Oxidative Stress in Humans. Oxid. Med. Cell Longev..

